# Heritability and genetic correlations of plasma metabolites of pigs with production, resilience and carcass traits under natural polymicrobial disease challenge

**DOI:** 10.1038/s41598-021-99778-9

**Published:** 2021-10-19

**Authors:** E. Dervishi, T. Yang, M. K. Dyck, J. C. S. Harding, F. Fortin, PigGen Canada, J. Cheng, J. C. M. Dekkers, G. Plastow

**Affiliations:** 1 Livestock Gentec, Department of Agriculture, Food and Nutritional Science, Faculty of Agricultural, Life and Environmental Sciences, University of Alberta, Edmonton, AB Canada; 2 Department of Large Animal Clinical Sciences, University of Saskatchewan, Saskatoon, SK Canada; 3 Centre de Developpement du Porc du Quebec Inc. (CDPQ), Quebec City, QC Canada; 4 PigGen Canada Research Consortium, Guelph, ON N1H4G8 Canada; 5 Department of Animal Science, Iowa State University, Ames, IA 50011 USA; 6 Fast Genetics, 8 - 4003 Millar Avenue, Saskatoon, SK S7K 2K6 Canada; 7 Genesus Genetics, 101 2nd St, Oakville, MB R0H 0Y0 Canada; 8 Hypor Canada, Spoorstraat 69, 5831CK Boxmeer, The Netherlands; 9 Topigs Canada, 20 South Landing, Unit 1, Oak Bluff, MB R4G 0C4 Canada; 10 DNA Genetics, 4438 Old Mill Court, Columbus, NE 68601 USA; 11 ALPHAGENE, 2200, Pratte Avenue, Saint-Hyacinthe, QC J2S 4B6 Canada; 12 Alliance Genetics, PO Box 24039, Edward RPO 107 Edward St., St. Thomas, ON N5P 1Y0 Canada

**Keywords:** Genetics, Animal breeding, Genomics, Metabolomics

## Abstract

Metabolites in plasma of healthy nursery pigs were quantified using nuclear magnetic resonance. Heritabilities of metabolite concentration were estimated along with their phenotypic and genetic correlations with performance, resilience, and carcass traits in growing pigs exposed to a natural polymicrobial disease challenge. Variance components were estimated by GBLUP. Heritability estimates were low to moderate (0.11 ± 0.08 to 0.19 ± 0.08) for 14 metabolites, moderate to high (0.22 ± 0.09 to 0.39 ± 0.08) for 17 metabolites, and highest for l-glutamic acid (0.41 ± 0.09) and hypoxanthine (0.42 ± 0.08). Phenotypic correlation estimates of plasma metabolites with performance and carcass traits were generally very low. Significant genetic correlation estimates with performance and carcass traits were found for several measures of growth and feed intake. Interestingly the plasma concentration of oxoglutarate was genetically negatively correlated with treatments received across the challenge nursery and finisher (− 0.49 ± 0.28; *P* < 0.05) and creatinine was positively correlated with mortality in the challenge nursery (0.85 ± 0.76; *P* < 0.05). These results suggest that some plasma metabolite phenotypes collected from healthy nursery pigs are moderately heritable and genetic correlations with measures of performance and resilience after disease challenge suggest they may be potential genetic indicators of disease resilience.

## Introduction

Metabolites are small molecules with molecular size < 1.5 kDa and metabolomics is the study of these small molecules to provide a more detailed and comprehensive understanding of how cells function. Metabolites are involved in cellular metabolism, such as energy conversion, growth, signaling, and stimulatory and inhibitory effects on enzymes^1,2^ and can, therefore, be considered as a bridge between genotype and phenotype. Metabolomics has been used in pharmacology studies, drug development, food technology, toxicology, plant biotechnology, and human health. In the latter case, metabolomics has been used successfully for disease diagnosis and determination of disease state, biomarker discovery, and risk determination^3^. Metabolite testing is increasingly gaining attention in animal science, including in animal genetics, animal health, and milk and meat quality^4^. Applications in livestock include discovery of disease biomarkers in dairy cows^5,6^, animal health^7^, growth performance^8^, feed efficiency in beef cattle and pigs^9,10^, and swine diseases^11^.

The large impact of infectious disease in swine on animal welfare and productivity has increased interest in selection for pigs that are less susceptible to pathogens and that are more disease resilient, which has been defined as the ability to maintain relatively undiminished performance levels when exposed to disease^12,13^. It has been proposed that resilience can be an effective approach to select for both disease resistance and tolerance in animals^13^. Results from studies with dairy cows, beef cattle, pigs including human medicine^5,8–10^, suggest that metabolomics can be useful in helping increase understanding of the processes involved in disease resilience. In addition, metabolomics offers the potential to identify new phenotypes or traits that can be used for selection of resilient animals. There is evidence in the literature that blood metabolites in humans and cattle are heritable^14–17^. Given the similarities that exist between porcine and human, blood metabolites in porcine might be expected to be heritable.

Heritability is a prerequisite for the use of a trait in breeding strategies and, ideally, indicators of resilience should be expressed in healthy animals, such that they can be measured in high-health nucleus farms to provide prediction of resilience in commercial environments^18^. It is therefore important to understand the genetic architecture of these potential new phenotypes.

This study is part of a larger project entitled “Phenomics for genetic and genome-enabled improvement of resilience in pigs”, which had identification of predictors of resilience on young healthy pigs as one of its main objectives. The project uses a natural polymicrobial disease challenge model^18^ with collection of resilience related traits, including average daily gain (ADG), feed intake and feed intake duration (ADFI and ADFD), number of individual health treatments (nTRT) and mortality, residual feed intake (RFI), and feed conversion ratio (FCR). In addition, new resilience traits based on day-to-day variation in feeding patterns were proposed by Putz et al^18^. The overall purpose of the current study was to use data and plasma samples collected on healthy pigs prior to pathogen/disease challenge to estimate heritability of 44 metabolites and 2 amino acid indices, the phenotypic and genetic parameters of plasma metabolite concentration in relation to their subsequent performance, disease resilience, and carcass traits under the natural disease challenge.

## Results

### Heritability

Table 1 summarizes estimates of heritabilities and variance components of 44 metabolites measured in plasma of young healthy pigs and two amino acid indexes. Heritability estimates for 13 metabolites were negligible, zero or not estimable. Metabolites with zero or negligible heritability were: glycerol, creatine, l-arginine, 3-hydroxybutyric acid, ethanol, formate, acetoacetate, succinate, acetone, methanol and l-acetylcarnitine. Among the 33 heritable metabolites, 14 showed low estimates of heritability, ranging from 0.11 ± 0.08 to 0.19 ± 0.08, while 17 metabolites had moderately high estimates of heritability, ranging from 0.22 ± 0.09 to 0.42 ± 0.08. Finally, two metabolites, i.e. l-glutamic acid and hypoxanthine, had the highest estimates of heritability, 0.41 ± 0.09 and 0.42 ± 0.08, respectively. Low and moderate estimates of heritability were obtained for two amino acid indexes, i.e. ketogenic amino acids (ketoAA) (0.23 ± 0.08) and branched-chain amino acids (BCAA) (0.19 ± 0.08).

**Table 1:** Estimates and standard errors (SE) of heritability and litter effects, phenotypic variance SD and variance due to pen (σ^2^_P_).

Metabolite	Heritability (SE)	Litter (SE)	Phenotypic SD (SE)	σ^2^P (SE)
l-Glutamic acid	0.42 (0.08)***	0.04 (006)	110.73 (3.35)	7023.2 (913.27)
Hypoxanthine	0.41 (0.09)***	0.15 (0.07)	24.02 (0.75)	337.13 (45.64)
Dimethylglycine	0.39 (0.08)***	0.07 (0.06)	3.46 (0.10)	7.34 (0.93)
l-Glutamine	0.38 (0.08)***	0.09 (0.06)	107.41 (3.23)	7045.3 (839.46)
l-Glycine	0.36 (0.08)***	0.10 (0.06)	367.02 (10.82)	84,876 (10,157)
Oxoglutarate	0.34 (0.09)***	0.07 (0.06)	18.8 (0.56)	231.34 (27.88)
Creatinine	0.34 (0.08)***	0.16 (0.06)	12.49 (0.37)	102.65 (11.78)
l-Ornithine	0.33 (0.08)***	0.07 (0.06)	36.36 (1.07)	886.72 (102.55)
Betaine	0.31 (0.08)***	0.16 (0.06)	28.09 (0.83)	537.44 (60.07)
Citric acid	0.30 (0.09)***	0.08 (0.05)	50.04 (1.47)	1745.7 (198.72)
Pyruvic acid	0.29 (0.09)***	0.22 (0.06)	69.58 (2.09)	3430.1 (395.32)
l-Lysine	0.27 (0.08)***	0.20 (0.05)	71.61 (2.12)	3699.5 (409.92)
l-Histidine	0.25 (0.08)***	0.10 (0.06)	9.81 (0.29)	73.02 (9.89)
l-Methionine	0.24 (0.09)*	0.12 (0.05)	16.35 (0.49)	202.36 (25.96)
ketoAA	0.23 (0.08)**	0.18 (0.05)	83.74 (2.46)	5344.9 (563.51)
l-Alpha-aminobutyric acid	0.23 (0.08)**	0.08 (0.05)	0.23 (0.06)	0.14 (1.00)
d-Glucose	0.22 (0.08)**	0.08 (0.05)	961.23 (27.42)	714,670 (70,966)
l-Aspartate	0.22 (0.08)***	0	6.93 (0.19)	36.9 (72.04)
Isobutyric acid	0.22 (0.09)**	0.11 (0.05)	1.86 (0.05)	3.37 (18.58)
l-Alanine	0.19 (0.08)*	0.03 (0.05)	194.36 (5.62)	30,447 (3014)
BCAA	0.19 (0.08)**	0.08 (0.05)	73.98 (2.10)	4419.5 (424.33)
l-Leucine	0.19 (0.08)**	0.08 (0.05)	25.08 (0.72)	508.72 (50.18)
l-Serine	0.18 (0.08)**	0.14 (0.05)	43.41 (1.23)	1530.2 (148.71)
l-Tyrosine	0.17 (0.08)**	0.09 (0.05)	16.11 (0.45)	214.17 (30.42)
2-Hydroxybutyrate	0.15 (0.07)**	0.09 (0.05)	0.22 (0.06)	0.48 (24.13)
l-Proline	0.15 (0.08)*	0.02 (0.05)	57.66 (1.64)	2796.7 (271.44)
l-Valine	0.14 (0.08)*	0.12 (0.05)	40.63 (1.14)	1411.3 (136.99)
l-Lactic acid	0.14 (0.08)*	0.12 (0.05)	2303.4 (65.34)	4,530,800 (436,890)
l-Phenylalanine	0.13 (0.07)*	0.07 (0.05)	12.21 (0.34)	129.68 (30.07)
l-Asparagine	0.14 (0.07)*	0.08 (0.05)	14.55 (0.40)	182.42 (27.66)
l-Threonine	0.12 (0.07)*	0.20 (0.05)	169.69 (4.77)	25,205 (2237.4)
l-Isoleucine	0.11 (0.08)*	0.17 (0.05)	22.65 (0.63)	456.54 (118.83)
3-Methy l-2-oxovaleric acid	0.11 (0.08)*	0.12 (0.05)	2.03 (0.05)	5.31 (60.02)
Glycerol	0.09 (0.08)	0.03 (0.05)	46.22 (1.29)	1934.9 (3637.9)
Creatine	0.07 (0.04)	0.01 (0.04)	119.35 (3.28)	12,972 (1068.3)
l-Arginine	0.07 (0.07)	0.06 (0.04)	13.29 (0.36)	164.73 (27.47)
3-Hydroxybutyric acid	0.06 (0.06)	0.02 (0.04)	0.21 (0.006)	0.07 (33.2)
Ethanol	0.06 (0.07)	–	0.23 (0.06)	1.99 (2166.8)
Formate	0.03 (0.01)	0.05 (0.02)	0.21 (0.006)	7.44 (2.37)
Acetoacetate	0.03 (0.07)	0.09 (0.04)	1.80 (0.04)	0.60 (261.57)
Succinate	0.02 (0.06)	0.11 (0.04)	8.74 (0.23)	75.08 (65.26)
Acetone	0	0.02 (0.04)	1.02 (0.03)	0.69 (587.11)
Methanol	0	0	28.23 (0.75)	7895.6 (631,140)
l-Acetylcarnitine	0	0	–	28.11 (0.00001)
1-Methylhistidine	–	–	3.24 (0.09)	7.44 (0.90)
2-Hydroxyisovalerate	–	–	–	–

Branched-chain amino acids (BCAA) was calculated as the sum of l-leucine, l-isoleucine and l-valine and ketogenic amino acids (ketoAA) was calculated as the sum of l-lysine and l-leucine. Significance of the heritability is indicated as ***, **, *, corresponding to *P* < 0.001, *P* < 0.01 and *P* ≤ 0.05. “–” indicates not estimable.

### Phenotypic correlations

In general, phenotypic correlations of the metabolites with performance, resilience, and carcass traits were small (Supplementary Figure S1a). However, there were some significant phenotypic correlations. l-Glutamine (0.12 ± 0.04), l-ornithine (0.12 ± 0.04), betaine (0.18 ± 0.04), citric acid (0.21 ± 0.04), l-lysine (0.13 ± 0.04) and l-methionine (0.15 ± 0.04) showed positive phenotypic correlation estimates with quarantine nursery ADG (qNurADG) (*P* < 0.05), while creatinine (− 0.21 ± 0.04), l-histidine (− 0.20 ± 0.04) and isobutyric acid (− 0.22 ± 0.04) showed negative phenotypic correlation estimates with qNurADG (P < 0.05). In addition, l-glutamic acid showed positive phenotypic correlation estimates with ADFI (0.11 ± 0.04; *P* < 0.05) and l-aspartate showed positive phenotypic correlation estimates with ADFD (0.18 ± 0.06; *P* < 0.05). None of the phenotypic correlations of metabolites with challenging nursery ADG (cNurADG), finisher ADG (FinADG), treatment, and mortality rates, and carcass traits were significant (Supplementary Table S1; *P* > 0.05).

Moreover, we estimated the phenotypic correlations between the metabolites that are involved in the same pathway (glycine, serine, alanine and threonine metabolism) and the results showed that betaine was positively correlated with dimethylglycine and l-serine, and l-serine was positively correlated with l-methionine (Supplementary Table S2). In addition, betaine did have significant positive phenotypic correlations with l-glycine (Supplementary Table S3; *P* < 0.05).

### Genetic correlations

**Figure 1:**
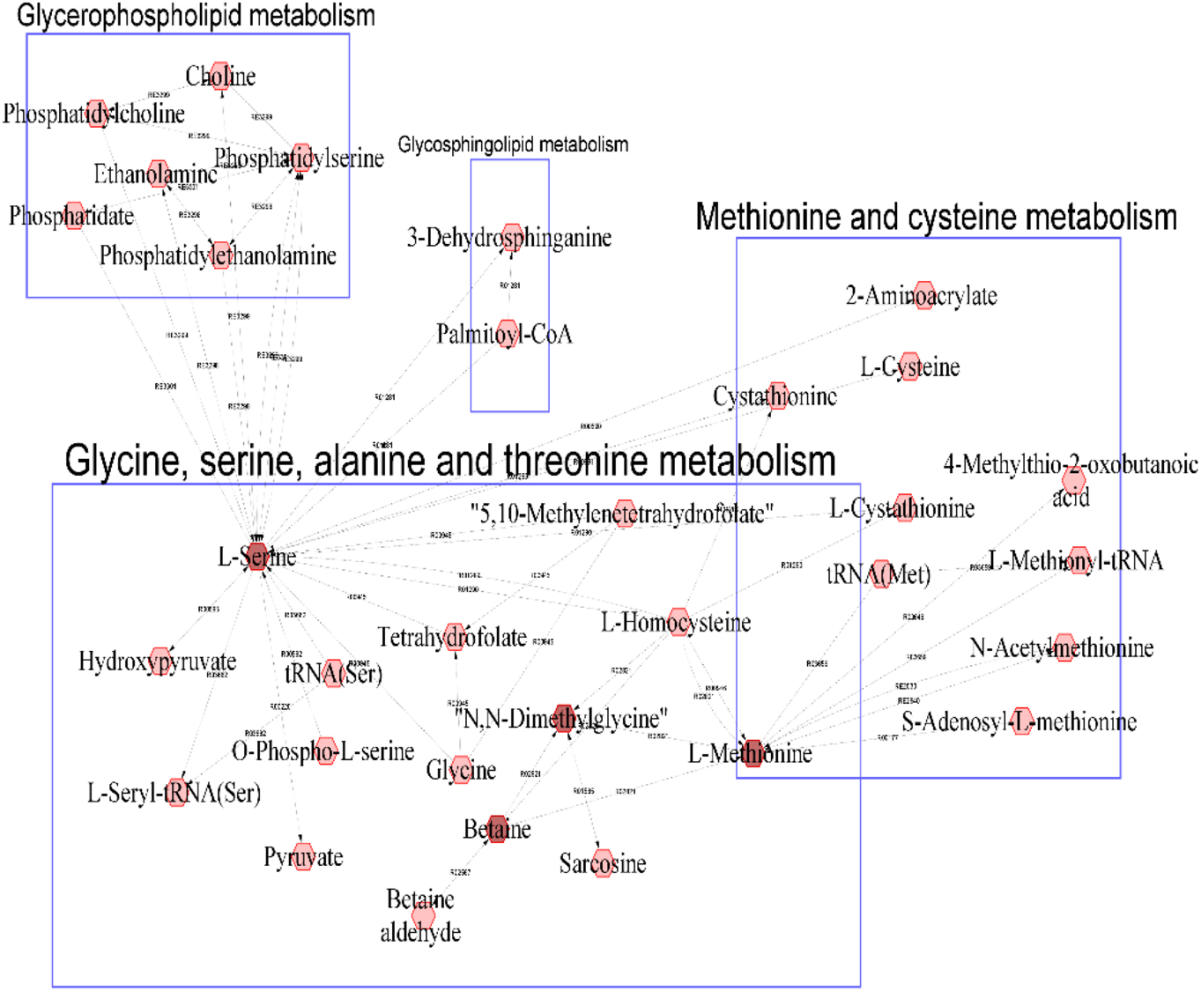
A compound network of metabolites that had significant (*P* < 0.05) genetic correlations with average daily gain in the quarantine nursery. Input metabolites are shown in dark red color and chemical reactions are represented as edges.

Genotypic correlations of the metabolites with performance, resilience, and carcass traits were larger than phenotypic correlation (Supplementary Figure S1b) however with larger SE. Estimates of genetic correlation between metabolites and ADG in the three phases, qNurADG, cNurADG and FinADG are provided in Table 2. Overall, estimates of genetic correlations among plasma metabolites and ADG in the three phases were very low, with high SE. However, some metabolites showed significant correlation estimates, with the largest negative genetic correlation between plasma creatinine and qNurADG (− 0.60 ± 0.18). Dimethylglycine, betaine, L-methionine and L-serine showed positive genetic correlation estimates with qNurADG. Metabolites that were positively correlated with qNurADG are visualized in a compound network in Fig. 1. The metabolites that were genetically positively correlated with qNurADG are involved in metabolic pathways, including the glycine and serine, methionine and cysteine, glycerophospholipid, and glycosphingolipid pathways. We estimated the genetic correlations between the metabolites that are involved in the same pathway (glycine, serine, alanine and threonine metabolism) and the results showed no significant genetic correlations between these metabolites (Supplementary Table S2).

**Table 2:** Estimates (SE in parentheses) of genetic correlations between the most heritable metabolites and average daily gain in three stages, i.e. the quarantine nursery, the challenge nursery, and the finisher.

Metabolite	Quarantine nursery	Challenge nursery	Finisher
l-Glutamic acid	0.14 (0.16)	0.06 (0.20)	0.72 (0.21)**
Hypoxanthine	0.09 (0.17)	− 0.22 (0.20)	0.18 (0.22)
Dimethylglycine	0.28 (0.17)*	0.06 (0.20)	0.36 (0.21)^x^
l-Glutamine	0.17 (0.16)	0.16 (0.21)	0.52 (0.26)
l-Glycine	0.23 (0.17)	0.08 (0.21)	0.08 (0.22)
Oxoglutarate	− 0.07 (0.18)	− 0.15 (0.23)	0.46 (0.24)*
Creatinine	− 0.60 (0.18)**	0.07 (0.22)	–
l-Ornithine	0.16 (0.18)	− 0.15 (0.24)	− 0.04 (0.25)
Betaine	0.39 (0.17)*	− 0.36 (0.23)^x^	0.19 (0.24)
Citric acid	0.32 (0.20)^x^	− 0.41 (0.25)^x^	–
Pyruvic acid	− 0.32 (0.22)^x^	− 0.34 (0.32)	0.12 (0.27)
l-Lysine	0.29 (0.21)^x^	0.05 (0.25)	0.15 (0.29)
l-Histidine	− 0.29 (0.26)	0.10 (0.29)	0.14 (0.31)
l-Methionine	0.45 (0.32)*	− 0.12 (0.55)	− 0.17 (0.34)
KetoAA	0.30 (0.24)	0.01 (0.30)	0.13 (0.35)
l-Alpha-aminobutyric acid	0.39 (0.44)	− 0.13 (0.91)	− 0.11 (0.30)
d-glucose	0.22 (0.19)	0.26 (0.39)	− 0.21 (0.23)
Isobutyric acid	− 0.21 (0.24)	0.05 (0.32)	− 0.11 (0.32)
l-Aspartate	− 0.07 (0.02)	0.13 (0.02)	− 0.21 (3.04)
l-sSrine	0.54 (0.33)*	0.11 (0.31)	− 0.04 (0.92)

Significance of genetic correlations are indicated in bold as: **, *, ^*x*^ corresponding to *P* < 0.01, *P* ≤ 0.05 and 0.05 < *P* < 0.10 respectively; “–” indicates not estimable.

No significant genetic correlations were estimated between metabolites and cNurADG. Only two metabolites namely: l-glutamic acid and oxoglutarate were estimated to have significant (positive) genetic correlations with FinADG.

**Figure 2:**
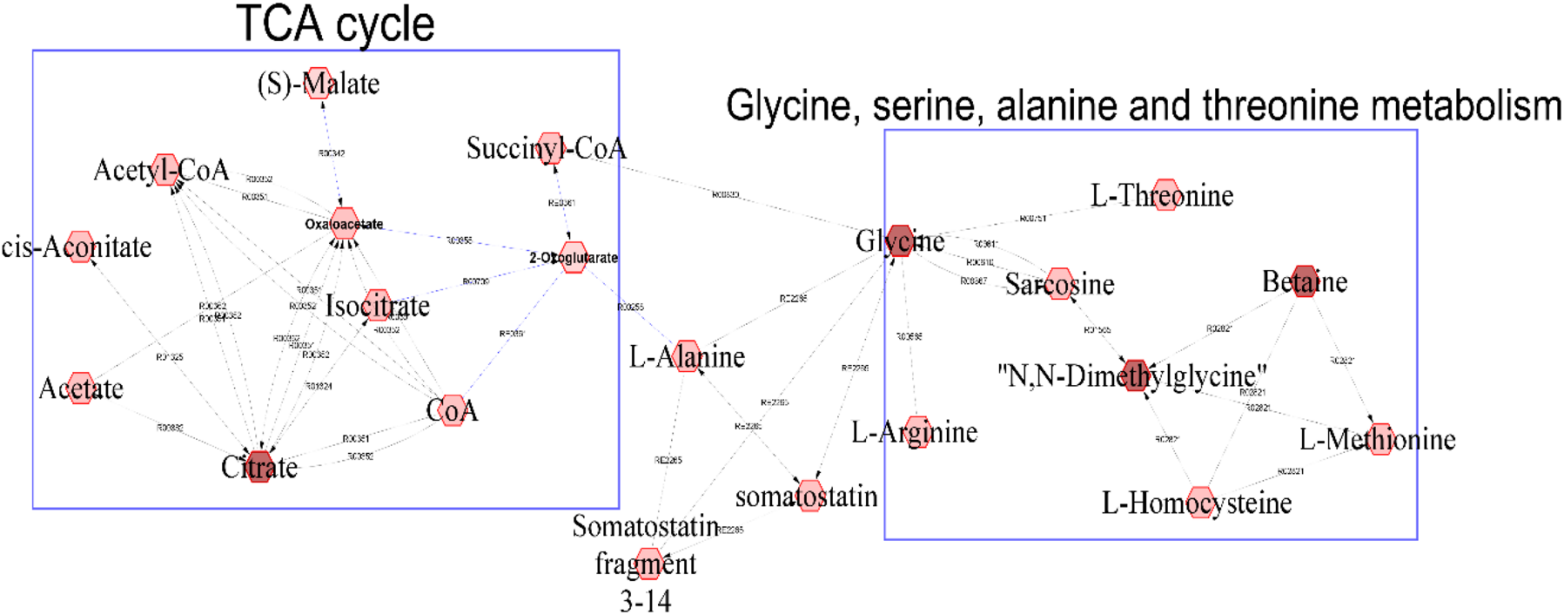
A compound network of metabolites that had significant (*P* < 0.05) genetic correlation with average daily feed duration. Input metabolites are shown in dark red color and chemical reactions are represented as edges.

Table 3 shows estimates of genetic correlations of metabolites with the feed intake traits ADFI, ADFD, FCR, and RFI. l-Glutamic acid was positively correlated with ADFI (*P* < 0.001), while several other metabolites, including dimethylglycine and l-aspartate, tended to be positively genetically correlated with ADFI (0.05 < *P* < 0.1). Four metabolites that were positively correlated with ADFD (Table 3; *P* < 0.05): dimethylglycine, l-glycine, betaine and citric acid were visualized in a compound network (Fig. 2). These metabolites are involved in metabolic pathways such as the TCA cycle and the glycine and serine, methionine, and cysteine pathways but they were not significantly genetically correlated with each other (Supplementary Table S3).

**Table 3:** Estimates of genetic correlations (SE in parentheses) between the most heritable metabolites and average daily feed intake (ADFI), duration (ADFD), feed conversion ratio (FCR) and residual feed intake (RFI).

Metabolite	ADFI	ADFD	FCR	RFI
l-Glutamic acid	0.62 (0.18)***	0.62 (0.70)	0.01 (0.20)	− 0.03 (0.02)
Hypoxanthine	0.17 (0.19)	0.39 (0.16)	− 0.20 (0.27)	0.06 (0.15)
Dimethylglycine	0.31 (0.18)^x^	0.33 (0.15)**	− 0.40 (0.26)^x^	0.09 (0.20)
l-Glutamine	0.42 (0.69)	0.48 (0.68)	0.47 (0.24)*	− 0.1 (0.15)
l-Glycine	0.19 (0.19)	0.30 (0.14)*	0.26 (0.28)	0.12 (0.16)
Oxoglutarate	0.34 (0.22)	0.29 (0.17)	− 0.01 (0.31)	− 0.17 (0.32)
Creatinine	0.58 (1.03)	0.14 (0.15)	− 0.31 (0.26)	− 0.14 (0.16)
l-Ornithine	0.10 (0.21)	0.25 (0.17)	0.01 (0.32)	0.34 (0.21)
Betaine	− 0.25 (0.46)	0.53 (0.16)***	0.12 (0.33)	0.16 (0.17)
Citric acid	0.1 (0.39)	0.42 (0.19)*	0.37 (0.29)	–
Pyruvic acid	0.07 (0.24)	0.14 (0.19)	− 0.03 (0.33)	0.21 (0.20)
l-Lysine	0.26 (0.26)	− 0.09 (0.37)	0.23 (0.36)	0.29 (0.19)
l-Histidine	0.04 (0.26)	− 0.42 (0.42)	− 0.16 (0.35)	0.19 (0.29)
l-Methionine	− 0.12 (0.28)	0.22 (0.26)	–	− 0.24 (0.30)
KetoAA	0.23 (0.29)	0.05 (0.23)	–	0.48 (1.05)
l-Alpha-aminobutyric acid log	0.05 (0.27)	0.12 (0.28)	0.63 (0.51)	− 0.07 (0.26)
d-Glucose	− 0.07 (0.26)	− 0.15 (0.30)	0.04 (0.39)	0.05 (0.19)
Isobutyric acid	− 0.28 (0.28)	− 0.38 (0.29)^x^	− 0.13 (0.61)	− 0.38 (0.27)*
l-Aspartate	0.49 (0.37)^x^	0.52 (0.61)	− 0.59 (0.58)	− 0.22 (0.32)

Significance of genetic correlations are indicated in bold as: ***, **, *, ^x^ corresponding to *P* < 0.001, *P* < 0.01, *P* ≤ 0.05 and 0.05 < *P* < 0.10 respectively. “–” indicates not estimable.

In addition, the l-glutamine was positively correlated at the genotypic level with FCR (*P* < 0.05) and isobutyric acid was negatively correlated with RFI (*P* < 0.05).

Estimates of genetic correlations of metabolites with carcass traits are presented in Table 4. The only significant estimate was of citric acid with carcass back fat (CBF) (*P* < 0.05).

**Table 4:** Estimates of genetic correlations (SE in parentheses) between the most heritable metabolites and carcass traits: carcass weight (CWT), backfat (CBF) and loin depth (CLD), lean yield (LYLD) and dressing percentage (DRS).

Metabolite	CWT	CBF	CLD	LYLD	DRS
l-Glutamic acid	− 0.11 (0.33)	0.12 (0.5)	− 0.20 (0.18)	− 0.12 (0.14)	− 0.21 (0.31)
Hypoxanthine	− 0.29 (0.33)	0.26 (0.16)	− 0.22 (0.19)	− 0.27 (0.16)^x^	− 0.25 (0.25)
Dimethylglycine	0.24 (0.34)	0.13 (0.16)	− 0.23 (0.19)	–	− 0.01 (0.25)
l-Glutamine	0.17 (0.33)	0.22 (0.15)	0.08 (0.19)	− 0.18 (0.15)	0.11 (0.24)
l-Glycine	0.003 (0.34)	0.26 (0.15)^x^	− 0.21 (0.19)	− 0.24 (0.16)	0.16 (0.26)
Oxoglutarate	− 0.34 (0.40)	0.44 (0.80)	− 0.31 (0.21)	− 0.08 (0.17)	− 0.39 (0.27)
Creatinine	0.27 (0.37)	0.46 (0.42)	0.02 (0.20)	− 0.30 (0.17)^x^	0.45 (0.30)
l-Ornithine	− 0.51 (0.50)	0.16 (0.18)	− 0.18 (0.22)	− 0.16 (0.18)	− 0.26 (0.27)
Betaine	− 0.24 (0.36)	0.08 (0.17)	− 0.09 (0.45)	− 0.17 (0.19)	− 0.20 (0.28)
Citric acid	0.41 (0.39)	0.34 (0.18)*	− 0.70 (0.48)^x^	0.16 (0.04)	0.19 (0.29)
Pyruvic acid	− 0.16 (0.82)	0.04 (0.14)	0.40 (1.12)	0.003 (0.15)	0.16 (1.76)
l-Lysine	0.79 (0.06)	0.72 (0.7)	− 0.20 (2.35)	− 0.74 (1.06)	− 0.01 (0.33)
l-Histidine	0.40 (0.13)	0.40 (1.52)	0.02 (2.66)	− 0.31 (2.87)	− 0.57 (1.14)
l-Methionine	0.28 (1.35)	0.76 (0.10)^x^	− 0.68 (0.74)	− 0.79 (0.11)^x^	0.22 (0.67)
KetoAA	0.76 (0.06)	0.59 (1.06)	− 0.15 (2.20)	− 0.81 (0.71)	0.02 (0.36)
l-Alpha-aminobutyric acid log	0.15 (0.51)	0.02 (0.25)	− 0.03 (0.26)	− 0.03 (0.22)	0.12 (0.66)
D-glucose	− 0.40 (0.49)	− 0.04 (0.22)	− 0.06 (030)	0.03 (0.22)	− 0.24 (0.37)
Isobutyric acid	0.90 (1.74)	− 0.12 (0.24)	0.25 (0.79)	0.10 (0.25)	0.46 (0.69)
l-Aspartate	− 0.04 (0.79)	0.16 (0.30)	− 0.33 (0.36)	− 0.19 (0.28)	− 0.47 (0.87)

Significance of genetic correlations are indicated in bold as: *, ^x^ corresponding to *P* ≤ 0.05 and 0.05 < *P* < 0.10 respectively. “–” indicates not estimable.

Finally, only oxoglutarate had a significant negative genetic correlation estimate with the number of treatments across the challenge nursery and finisher (Table 5; *P* < 0.05), while creatinine had a significant positive genetic correlation with mortality in the challenge nursery (Table 6; *P* < 0.05).

**Table 5:** Estimates of genetic correlations (SE in parentheses) between the most heritable metabolites and number of treatments in the challenge nursery, finisher, and across the nursery and finisher.

Metabolite	Challenge nursery	Finisher	Nursery finisher
l-Glutamic acid	0.22 (0.23)	− 0.53 (0.86)	− 0.45 (0.97)
Hypoxanthine	0.06 (0.24)	− 0.47 (0.93)	− 0.24 (0.35)
Dimethylglycine	0.04 (0.24)	0.11 (0.77)	–
l-Glutamine	− 0.27 (0.26)	− 0.43 (0.88)	− 0.08 (0.06)
l-Glycine	− 0.08 (0.26)	0.08 (0.87)	–
Oxoglutarate	− 0.11 (0.26)	− 0.90 (1.28)	− 0.49 (0.28)*
Creatinine	− 0.32 (0.29)	− 0.18 (0.70)	− 0.34 (0.29)
l-Ornithine	− 0.09 (0.31)	− 0.71 (1.27)	− 0.24 (0.36)
Betaine	0.04 (0.26)	0.05 (1.48)	0.19 (0.33)
Citric acid	− 0.09 (0.28)	− 0.36 (1.01)	− 0.10 (0.32)
Pyruvic acid	− 0.30 (0.32)	0.25 (1.16)	− 0.16 (0.31)
l-Lysine	− 0.19 (0.31)	− 0.53 (1.21)	− 0.17 (0.40)
l-Histidine	0.05 (0.31)	− 0.37 (1.11)	− 0.34 (0.77)
l-Methionine	− 0.07 (0.52)	− 0.08 (1.27)	0.43 (0.51)
KetoAA	− 0.03 (0.35)	− 0.25 (1.45)	0.03 (0.53)
l-Alpha-aminobutyric acid log	0.41 (0.40)	0.11 (1.09)	0.48 (0.62)
d-Glucose	− 0.14 (0.38)	− 0.04 (0.02)	–
Isobutyric acid	0.14 (0.37)	− 0.82 (1.46)	− 0.44 (0.75)
l-Aspartate	0.06 (0.38)	0.58 (4.33)	− 0.12 (0.46)

Significance of genetic correlations are indicated in bold as: *, corresponding to *P* ≤ 0.05. “–” indicates not estimable.

**Table 6:** Estimates of genetic correlations (SE in parentheses) between the most heritable metabolites and mortality in the challenge nursery, finisher, and across the nursery and finisher.

Metabolite	Challenge nursery	Finisher	Nursery finisher
l-Glutamic acid	–	− 0.77 (1.00)	–
Hypoxanthine	0.12 (0.35)	− 0.67 (0.91)	− 0.83 (1.72)
Dimethylglycine	− 0.08 (0.55)	0.25 (0.59)	0.18 (0.69)
l-Glutamine	− 0.21 (0.44)	− 0.14 (0.59)	− 0.05 (1.03)
l-Glycine	0.11 (0.35)	0.28 (0.60)	0.24 (0.79)
Oxoglutarate	–	− 0.76 (1.51)	–
Creatinine	0.85 (0.76)*	− 0.12 (2.01)	− 0.04 (0.28)
l-Ornithine	0.01 (0.54)	–	− 0.30 (0.73)
Betaine	–	–	0.09 (0.76)
Citric acid	− 0.13 (8.36)	0.12 (0.58)	− 0.06 (0.84)
Pyruvic acid	0.12 (0.39)	− 0.25 (0.73)	− 0.33 (0.88)
l-Lysine	0.39 (0.40)	− 0.09 (1.06)	− 0.15 (1.54)
l-Histidine	0.68 (0.75)	− 0.35 (0.92)	− 0.17 (0.96)
l-Methionine	− 0.24 (0.98)	0.09 (0.87)	− 0.08 (0.72)
KetoAA	0.10 (0.87)	− 0.27 (1.04)	− 0.35 (1.21)
l-Alpha-aminobutyric acid log	0.24 (0.74)	0.33 (0.83)	0.39 (1.07)
d-Glucose	− 0.21 (0.51)	− 0.10 (0.78)	− 0.16 (1.04)
Isobutyric acid	0.08 (0.70)	− 0.21 (0.96)	− 0.22 (3.35)
l-Aspartate	− 0.50 (1.70)	–	–

Significance of genetic correlations are indicated in bold as: *, corresponding to *P* ≤ 0.05. “–” indicates not estimable.

## Discussion

The objectives of this study were to estimate (1) heritabilities of metabolites in plasma of young healthy pigs and (2) their genetic correlations with production, disease resilience, and carcass traits in growing pigs that were exposed to a natural polymicrobial disease challenge. The metabolomics samples were collected from healthy pigs at an average of 26 days of age in the quarantine nursery and the traits analyzed included those in the quarantine nursery, as well as in the challenge nursery and finisher, and carcass traits at slaughter. This study contributes to one of the main overarching objectives of the polymicrobial natural disease challenge model, which is to identify genetic predictors of resilience using samples from healthy pigs. Such predictors would be very useful for breeding for disease resilience, as they can be measured in high health genetic nucleus herds as indicator traits for disease resilience in commercial farms.

We measured the concentration of a panel of 44 metabolites in plasma, including amino acids, short chain fatty acids, sugars, alcohols, organic acids, amines, and TCA cycle intermediates, and urea cycle intermediates. Variation in metabolite concentration can be due to environmental effects, diet, gender, age, physiological conditions, and genetic effects. Literature indicates that in humans, approximately 50% of phenotypic differences in metabolite levels is due to genetics, but heritability estimates differ across metabolite classes^16^. Metabolites can be grouped into primary and secondary metabolites. Primary metabolites are directly involved in primary metabolic processes, such as normal growth, development, reproduction, and immune response, e.g. amino acids and products derived from glycolysis and the TCA cycle. Primary metabolites are highly conserved across species and serve as precursors for the synthesis of secondary metabolites^19^. For example, amino acids, in addition to being the building blocks of proteins, are also regulators of innate and adaptive immune responses in living cells. Many studies have demonstrated that glutamic acid, glutamine, histidine, methionine, leucine, isoleucine, and valine are functional regulators of macrophages, dendritic cells, and T-cells.^20–23^. Our results showed that 31 metabolites had low to moderate heritability and two metabolites had relatively high heritability (> 0.4). In a study of beef cattle, only 11 of 33 metabolites measured (29 in common with this study) were reported to be heritable^17^. Similar heritability estimates to Li et al.^17^ were reported here for betaine, creatinine, pyruvic acid and citric acid. In our study, other metabolites, such as ketone bodies (3-hydroxybutyric acid, acetoacetate and acetone), creatine, succinate, formate, and methylhistidine, showed negligible heritability estimates, suggesting that they are primarily influenced and manipulated by environmental effects such as diet, and/or age, health status etc.

Branched-chain amino acids, was calculated as the sum of l-leucine, l-isoleucine and l-valine and BCAA did not have higher heritability than the amino acids that contributed to the index.

Phenotypic correlations of metabolites with production, disease resilience, and carcass traits were generally very low. These results are in line with those reported previously in dairy cows by Buitenhuis et al.^15^. However, genetic correlation analyses found that dimethylglycine (0.28), betaine (0.39), l-methionine (0.45), and l-serine (0.54) were positively correlated with ADG in the quarantine nursery, while creatinine (− 0.60) was negatively correlated with this trait. These metabolites are involved in glycine, serine, alanine and threonine pathway, which suggests that this pathway might be a target to improve ADG in young healthy piglets. Indeed, supplementation with dimethylglycine has been shown to improve growth performance, significantly increasing total body weight gain and feed intake, and improving feed efficiency in low-birth-weight piglets^24^. We found that l-methionine (0.15), l-glutamine (0.12) and betaine (0.18), had positive phenotypic correlation estimates with qNurADG. Methionine supplementation has been reported to improve growth rate in nursery pigs^25^. Positive effects of methionine supplementation on intestine structure have also been observed, with greater average daily gain from days 7–14 of age and improved feed efficiency^25^.

Creatinine was genetically highly negatively correlated (− 0.60) with ADG in the quarantine nursery. Variation in creatinine concentration can arise as result of environmental and genetic factors. For example, phenotypic variation in creatinine concentration can be expected due to batch, transportation and fighting or re-grouping of animals. Creatinine is considered a waste product produced by muscles from the breakdown of creatine and is removed from the blood and released into urine by the kidneys. Typically, 95% of creatinine is found in muscle and an increase of creatinine concentration in blood is often considered as an indicator of kidney malfunction^27^. Conversion of muscle creatine into creatinine can reflect protein degradation^28^ and serum creatinine concentration has been proposed as an indicator of protein deposition^29^. Animals with low ADG are expected to have lower protein or muscle deposition and our results suggest that increased plasma creatinine concentration might be a genetic indicator of low ADG in healthy nursery piglets. In addition, creatinine was phenotypically negatively correlated with ADG of young healthy pigs (− 0.21). Moreover, creatinine was genetically positively correlated with mortality in the challenge nursery, suggesting that young healthy pigs that have higher plasma creatinine, genetically have lower ADG as healthy nursery pigs but are more likely to die when challenged by disease. Further research is necessary to validate creatinine as a potential genetic marker for ADG in healthy nursery pigs and as an early genetic indicator trait for mortality under disease.

Plasma oxoglutarate concentration in the quarantine nursery was genetically positively correlated with ADG in the finisher and negatively correlated with nTRT across the nursery and finisher. Moreover, l-glutamic acid showed positive genetic correlation estimates with ADG (0.72) and with ADFI (0.62) in the finisher. Oxoglutarate, also known as alpha-ketoglutarate, is a key organic acid of the TCA cycle and a source of glutamic acid and glutamine, and stimulates protein synthesis and inhibits protein degradation in muscles^30,31^. Positive effects of oxoglutarate on the protein synthesis and skeletal system have been reported in various farmed species, including turkeys^32^, pigs^33,34^, and sheep^35,36^. Our results suggest that young healthy pigs that had greater oxoglutarate and l-glutamic acid concentration, genetically have greater ADG in the finisher and received fewer health treatments across the challenge nursery and finisher. Interestingly, none of the metabolites were genetically correlated with ADG in the challenge nursery. For the data used here, Cheng et al.^37^ reported that the estimate of heritability of ADG was lower in the challenge nursery (0.19) than in the quarantine nursery (0.31) and in the finisher (0.30), which might have impacted the ability to accurately estimate the genetic correlations of metabolites with ADG in the challenge nursery.

Four metabolites (dimethylglycine, l-glycine, betaine and citric acid) had moderate to high positive genetic correlation estimates with ADFD (0.30–0.52). These metabolites are involved in the TCA cycle and in glycine, serine, alanine, and threonine metabolism. Interestingly, dimethylglycine and betaine were also positively correlated with ADG in the quarantine nursery, which suggests that pigs with greater dimethylglycine and betaine concentration genetically have greater ADG as young healthy pigs and might spend more time eating in the finisher stage.

A moderate positive genetic correlation (0.34) between citric acid and carcass backfat was estimated, which is in line with the fact that citric acid is involved in fatty acid metabolism. Citrate, the conjugated base of citric acid, is formed from oxaloacetate and acetyl-coenzyme A (acetyl-CoA) by citrate synthase. Once transported to the cytosol, citrate is converted to acetyl CoA and oxaloacetate. Acetyl-CoA is then converted to malonyl CoA and can be used as a substrate for fatty acid synthesis^38^. The genetic correlation observed suggests that young healthy pigs that have higher plasma content of citric acid genetically have higher carcass backfat when disease is present.

Interestingly, isobutyric acid was estimated to be negatively correlated, genetically, with RFI (− 0.38), which suggests that young healthy pigs that have higher plasma isobutyric acid content genetically have better feed efficiency under disease conditions. Isobutyric acid is a carboxylic or short chain fatty acid (SCFA) that is generated via microbial (gut) metabolism. Isobutyric acid has been described and quantified in the faeces of human, rats, horses, and pigs^39^. It has been reported that SCFAs such as acetic, propionic, and butyric acids, derive mostly from carbohydrates, while other SCFAs such as isobutyric acid are mostly derived from proteins^40^, specifically from degradation of branched-chain amino acids, and greater concentration of isobutyric acid could be indicative of better utilization of dietary protein by the microbiota^41^. Literature indicates that low RFI animals (efficient) have greater ileal isobutyric acid concentrations^42^. Thus, our results suggest that low RFI pigs genetically might have a better utilization of dietary protein by the microbiota. Further research is necessary to investigate the role of isobutyric acid as a potential genetic biomarker of RFI in pigs with or without disease.

In conclusion, the results suggest that some metabolic phenotypes measured in plasma of young healthy pigs are moderately heritable. The present work contributes to our understanding of the genetic parameters of plasma metabolite concentrations in young healthy pigs and their relationships with production, resilience, and carcass traits under disease. To the best of our knowledge, this is the first study to report estimates of heritabilities of plasma metabolite concentrations and of their genetic correlations with production, resilience, and carcass traits in pigs following polymicrobial disease challenge. Metabolites have the potential to be used in high health genetic nucleus herds as indicator traits for disease resilience in commercial farms. Further studies are warranted to validate the identified possible genetic indicators of resilience.

## Material and methods

### Animals, production, resilience and slaughter traits

This study was carried out in accordance with the Canadian Council on Animal Care guidelines (CCAC)^43^. All procedures were carried in accordance with the Animal Research: Reporting of In Vivo Experiments (ARRIVE) guidelines^44^. The Animal Care protocol was approved by the Animal Protection Committee of the Centre de Recherche en Sciences Animales de Deschambault (15PO283) and Animal Care and Use Committee at the University of Alberta (AUP00002227).

This study is part of a larger research project which investigates the underlying genetic mechanisms of disease resilience in grow-finisher pigs exposed to a natural polymicrobial disease challenge. The details of the polymicrobial challenge and phenotypes/traits collected were previously described in Putz et al.^18^, Cheng et al.^37^, and Bai et al.^45^. The challenge was established in late 2015 at the Centre de développement du porc du Québec inc (CDPQ) test station in Québec, Canada, with the aim to mimic a commercial farm with high disease pressure to maximize expression of genetic differences in resilience. For purpose of clarity, here we briefly describe the three phases of the experiment, which included a pre-challenge quarantine nursery (19 days on average beginning at 3 weeks of age), the challenge nursery (27 days on average), and lastly, the finishing phase (100 days on average). The natural disease challenge protocol was established by introducing naturally infected animals with known diseases into the challenge nursery and finisher barn at CDPQ. Some of the introduced pathogens included: viruses (PRRSV and swine influenza virus A), bacterial pathogens such as *Brachyspira hampsonii*, *Haemophilus parasuis*, *Mycoplasma hyopneumoniae*, *Salmonella enterica* serovar Typhimurium, and *Streptococcus suis*), and two parasites (*Ascaris suum* and *Cystoisospora suis*)^45^. Moreover, environmental enrichment (inedible point source objects) was applied in 50% of quarantine nursery pens in some of the batches, with the purpose to evaluate the impact of environmental enrichment on disease resilience. Pigs that received enrichment in the quarantine nursery, continued to receive enrichment in the challenge nursery and finisher^37^. Throughout the study feed was available ad libitum (the quarantine nursery, challenging nursery and finisher). In the quarantine nursery all pigs were fed the same commercial diet appropriate for pigs’ age and weight (Délice, Nourisson and Premier Age (Cie Alfred Couture ltée; Quebec, Canada)).

Production, resilience, and carcass traits were collected from a total of 3205 F1 crossbred (Landrace × Yorkshire) barrows. The phenotypes used in this study included: average daily gain recorded in the quarantine nursery, challenge nursery, and finisher stages. Finisher traits considered in the present study included feed intake and duration, feed conversion ratio, and residual feed intake. The number of parenteral treatments provided to individual pigs were also tabulated separately for the challenge nursery, finisher and combined challenge nursery and finisher as the number of treatments per 180 days. Mortality (0 = survived, 1 = died) in the challenge nursery, finisher, and across the nursery and finisher were also included^37^. Finally, carcass traits included: carcass weight, backfat, and loin depth, dressing percentage, and lean yield. Details of the recording and derivation of the resilience, production, and carcass traits can be found in Putz et al.^18^ and Cheng et al.^37^. Cheng et al.^37^ used two data sets for analysis of traits recorded in the finisher but in the current study we only use the data set that included phenotypic finisher data on pigs that survived to slaughter (survivor data), except for mortality. For the challenge nursery, phenotypes on all pigs were used. Details of the number of animals and traits considered here are described in Cheng et al.^37^. For purpose of brevity: data from 958 young healthy pigs were included in the present study for metabolomics analysis, nearly 3200 animals for nursery traits, around 2500 animals for finisher traits, and 2000 animals for carcass traits.

### Blood samples

For metabolomics analysis we used blood samples collected from 958 young healthy animals which were introduced in 15 batches of 60 or 75 pigs. Blood was collected from the jugular vein into K2 ethylenediaminetetraacetic acid (EDTA) tubes (BD Vacutainer, Blood Collection Tubes, United States), on all pigs in the quarantine nursery at an average age of 26 days, 5 days post-arrival from their farm of origin^45^. After collection, the blood samples were centrifuged at 3000 rpm at 4 °C for 10 min, plasma collected and immediately frozen and stored at − 80 °C and only thawed for the metabolomics analysis. Two weeks after the first sampling, pigs were transferred to the test station and naturally exposed to multiple pathogens, as described in Putz et al.^18^ and Bai et al.^45^.

All animals (n = 3205) were genotyped using a 650k Affymetrix Axiom Porcine Genotyping Array by Delta Genomics (Edmonton AB, Canada). Raw Affymetrix SNP data were processed by Delta Genomics, separately for each cycle, with the Axiom Analysis Suite, using all defaults. Details of genotyping and quality control are described in Cheng et al.^37^ and Bai et al.^45^. After quality control, a total of 417,443 SNPs in 3205 pigs remained and were used for analysis.

### Nuclear magnetic resonance spectroscopy and quality control

Forty-eight plasma metabolites were quantified using NMR, following established protocols at The Metabolomics Innovation Center at University of Alberta (TMIC), AB, Canada (https://www.metabolomicscentre.ca/).

Plasma samples were thawed on ice and a deproteinization step, involving ultra-filtration was performed as previously described^46^, in order to remove plasma macromolecules. Prior to filtration, 3 kDa cut-off centrifugal filter units (Amicon Microcon YM-3), were rinsed five times each with 0.5 mL of H_2_O and centrifuged (10,000 rpm for 10 min) to remove residual glycerol bound to the filter membranes. Aliquots of each plasma sample were then transferred into the centrifuge filter devices and spun (10,000 rpm for 20 min) to remove macromolecules (primarily protein and lipoproteins) from the sample. The filtrates were collected and the volumes for each sample were recorded. If the total volume of the sample was under 250 µL an appropriate amount of 150 mM KH_2_PO_4_ buffer (pH 7) was added and the dilution factor was annotated and taken into account in the analysis. Subsequently, 46.5 µL of a standard buffer solution (54% D_2_O:46% 1.75 mM KH_2_PO_4_ pH 7.0 v/v containing 5.84 mM DSS (2,2-dimethyl-2-silcepentane-5-sulphonate), 5.84 mM 2-chloropyrimidine-5 carboxylate, and 0.1% NaN_3_ in H_2_O) was added to the sample.

The plasma samples (250 µL) were transferred in 3 mm SampleJet NMR tubes for spectral analysis. All ^1^H-NMR spectra were collected on a 700 MHz Avance III (Bruker) spectrometer equipped with a 5 mm HCN Z-gradient pulsed-field gradient (PFG) cryoprobe. ^1^H-NMR spectra were acquired at 25 °C using the first transient of the NOESY pre-saturation pulse sequence (noesy1dpr), chosen for its high degree of quantitative accuracy^47^. All FID’s (free induction decays) were zero-filled to 250 K data points. The singlet produced by the DSS methyl groups was used as an internal standard for chemical shift referencing (set to 0 ppm) and for quantification all ^1^H-NMR spectra were processed and analyzed using an in-house version of the MAGMET automated analysis software package using a custom metabolite library. MAGMET allows for qualitative and quantitative analysis of an NMR spectrum by automatically fitting spectral signatures from an internal database to the spectrum^48^. Each spectrum was inspected by an NMR spectroscopist in order to minimize compound misidentification and misquantification.

Prior to statistical analysis, a quality control step was applied to the metabolite data. Four metabolites that were frequently (> 20%) below the limit of detection or with at least 20% missing values were removed from consideration. A total of 44 metabolites and two amino acid indexes remained in the dataset. Other missing values (15 data points) were replaced by the median value of each metabolite in the original data. First, we assessed the significance of each fixed (batch_,_ enrichment), covariable (age), and random effects (pen and litter) using linear regression models implemented in R statistical software^49^. The residuals of the model were plotted and visually inspected for the presence of outliers, which were excluded from the dataset. Data normalization (log10) of metabolite concentrations that were not normally distributed (2-hydroxybutyrate, ethanol, 3-hydroxybutyric acid, l-alpha-aminobutyric acid, methanol and creatine) was done prior to statistical analysis. Two indexes were also computed for statistical analysis: (1) branched-chain amino acids (BCAA), which was calculated as the sum of l-leucine, l-isoleucine and l-valine and (2) ketogenic amino acids (ketoAA), calculated as the sum of l-lysine and l-leucine. A summary of descriptive statistics of all metabolites after quality control is in Supplementary Table S4.

### Variance component analyses

Variance components were estimated by GBLUP using the BLUPF90 programs^50^. The general following mixed linear model was used to estimate the heritability of each metabolite:

$$Y_{ijk} = \, Batch_{i} + \, Age_{ijk} + \, Pen_{j} + \, Litter_{ijk} + \, u_{ijk} + \, e_{ijk}$$

where Y_*ijk*_ is the trait (metabolite); *Batch*_*i*_ is the fixed batch effect (*i* = 1, …, 15); *Age*_*ijk*_ is the covariate of age when the pig entered the quarantine nursery; *Pen*_*j*_ is the random effect of pen by batch corresponding the different phases (quarantine nursery, challenging nursery, or finisher), with *Pen*_*j*_ ~ N (0, σ^2^_P_) where σ^2^_P_ is pen variance; *Litter*_*ijk*_ is the litter environmental effect, with *Litter*_*ijk*_ ~ N (0, σ^2^_L_) where σ^2^_L_ is the litter environmental variance; *u*_*ijk*_ is the random additive genetic effect, with the vector ***u*** ~ N (**0**, **G**σ^2^_A_), where **G** is the genomic relationship matrix and σ^2^_A_ is the additive genetic variance; and *e*_*ijk*_ is the residual effect, with *e*_*ijk*_ ~ N (0, σ^2^_e_) where σ^2^_e_ is the residual variance. The genomic relationship matrix, **G**, was created separately for each of the seven companies supplying pigs using the software preGSf90^50^ and the method described by VanRaden^51^, and then combined into one **G** matrix, with genetic relationships between companies set to zero in order to focus on within-company variance components, as described by Cheng et al.^37^. For six metabolites namely: l-ornithine, l-leucine, l-valine, l-asparagine, 3-methyl 2-oxovaleric acid and formate, environment enrichment was included as a fixed effect because the effect of enrichment was significant (*P* ≤ 0.05).

For metabolites with heritability estimates greater than 0.20, genetic correlations with production, disease resilience, and carcass traits were estimated using bivariate models. For these traits, phenotypes from batches that were not measured for metabolites were also included. Models used for these traits were as described by Cheng et al.^37^.

Heritability was estimated as σ^2^_a_/ (σ^2^_a_ + σ^2^_L_ + σ^2^_e_) and the standard error (SE) for the heritability estimate was calculated according to the Monte-Carlo method suggested by Meyer and Houle^52^. The proportion of variance explained by sow or maternal effects, referring to effects that are common to individuals with the same mother, was estimated as σ^2^_L_/ (σ^2^_L_ + σ^2^_e_). Genetic correlations between two traits were estimated as the estimate of the genetic covariance from the bivariate analysis divided by the product of the genetic standard deviations for the two traits. Significance of estimates of heritabilities and genetic correlations were determined using likelihood ratio tests with 1 degree of freedom. For heritability estimates, the resulting P-values were divided by 2 because the estimates are restricted to be positive^53^.

### Network visualization

For exploration and visualization of the biochemical pathway that metabolites are involved in, the Metscape plugin^54^ in Cytoscape 3.8.2^55^ was used. The metabolites that had significant (*P* ≤ 0.05) genetic correlations with qNurADG and ADFD were used for pathway visualization. The file with the list of KEGG elements was loaded into Metscape to generate a compound network. Only metabolites with KEGG IDs were considered for compound network and pathway analysis. In a compound network, metabolites are represented as nodes and reactions are represented as edges. A compound node with an outgoing edge is a substrate, while a compound with an incoming edge is the product of a specific biochemical reaction. Finally, we estimated phenotypic and genetic correlations among metabolites that belong to the same pathway.

## Supplementary Information

Supplementary Figure S1.

Supplementary Table S1.

Supplementary Table S2.

Supplementary Table S3.

Supplementary Table S4.

## Data Availability

The data analyzed in this study are not publicly available, because the data were generated on samples from commercially owned animals, but they can be made available by the corresponding author on reasonable request.
