# Supplementary material for: Heritability and genetic correlations of plasma metabolites of pigs with production, resilience and carcass traits under natural polymicrobial disease challenge
Source: Sci Rep. 2021 Oct 19;11:20628. doi: 10.1038/s41598-021-99778-9 (PMC8526711; doi:10.1038/s41598-021-99778-9)

**Supplementary Figure S1**. Heatmap of estimates of a) phenotypic and b) genetic correlations between the most heritable metabolites and resilience and carcass traits. Red color indicates positive correlations and blue color indicates negative correlations. Average daily gain (ADG) in quarantine (qNurADG), challenge nursery (cNurADG), finisher (FinADG), average daily feed intake (ADFI) and duration (ADFD), feed conversion ratio (FCR), residual feed intake (RFI), number of treatments in the challenge nursery (nTrtcNur), finisher (nTrtFin), and across the nursery and finisher (nTrtcNurFin), mortality in the challenge nursery (MORcNur), finisher (MORFin), and across the nursery and finisher (MORcNurFin) carcass weight (CWT), backfat (CBF) and loin depth (CLD), lean yield (LYLD) and dressing percentage (DRS).


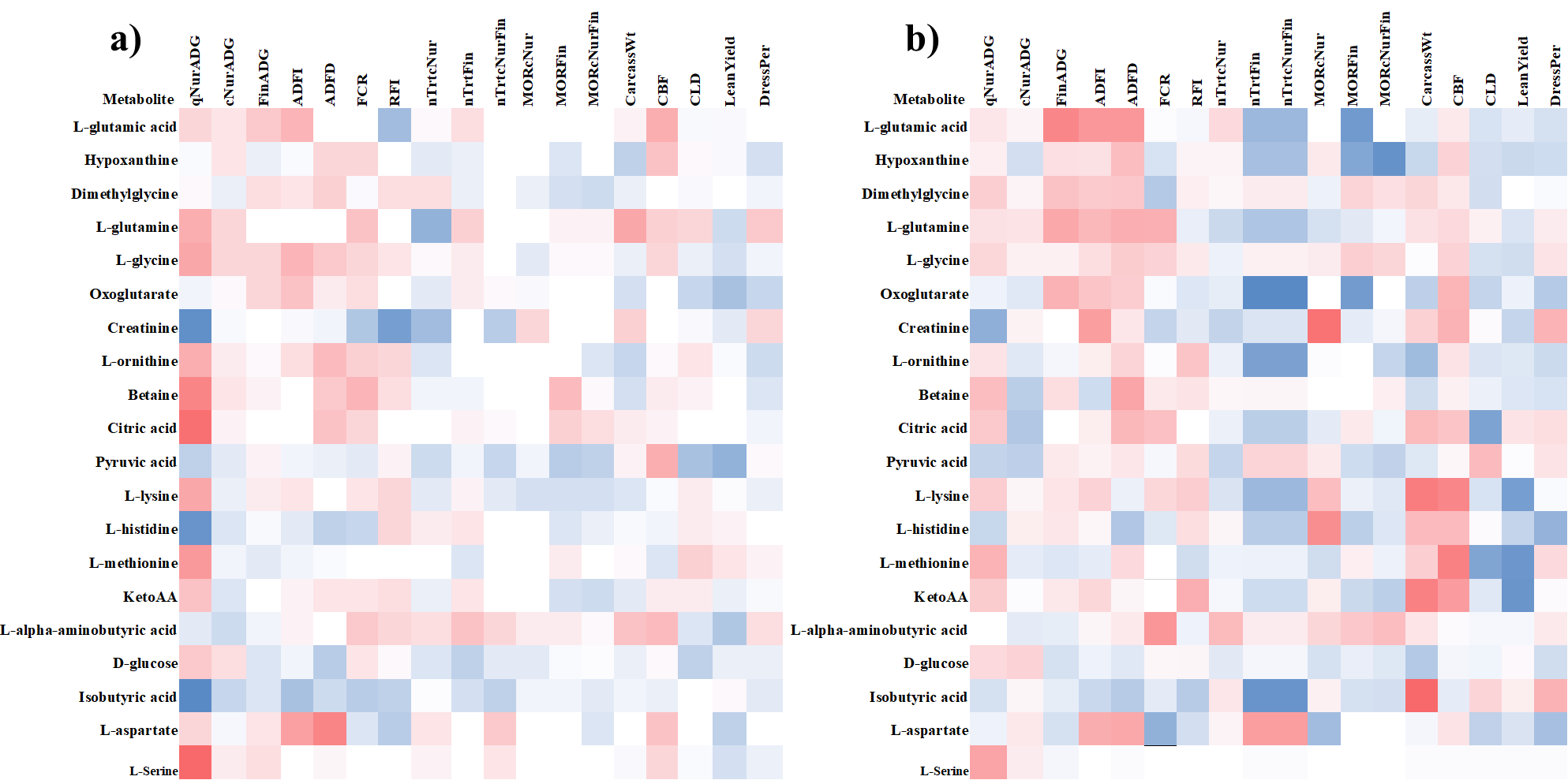

Supplement: Supplementary file 1 — Supplementary Figure S1. [file 41598_2021_99778_MOESM1_ESM.docx]
