# Supplementary material for: Heritability and genetic correlations of plasma metabolites of pigs with production, resilience and carcass traits under natural polymicrobial disease challenge
Source: Sci Rep. 2021 Oct 19;11:20628. doi: 10.1038/s41598-021-99778-9 (PMC8526711; doi:10.1038/s41598-021-99778-9)
Supplement: Supplementary file 3 — Supplementary Table S2. [file 41598_2021_99778_MOESM3_ESM.docx]

**Supplementary Table S2**. Estimates of genetic (above the diagonal) and phenotypic (below the diagonal) correlations (SE in parentheses) among betaine, dimethylglycine, L-serine and L-methionine. Significance of genetic and phenotypic correlations are indicated as: **, *, corresponding to P<0.01 and P<0.05 respectively; “-” indicates not estimable.

| **Metabolite** | Betaine | Dimethylglycine | L-serine | L-methionine |
| --- | --- | --- | --- | --- |
| Betaine | 1 | 0.28 (0.14) | -0.02 (0.48) | 0.07 (2.25) |
| Dimethylglycine | 0.40 (0.02)** | 1 | - | 0.21 (0.32) |
| L-serine | 0.35 (0.03)** | 0.13 (0.04) | 1 | -0.46 (1.43) |
| L-methionine | 0.23 (0.03) | 0.13 (0.03) | 0.19 (0.03)* | 1 |
