# Supplementary material for: Heritability and genetic correlations of plasma metabolites of pigs with production, resilience and carcass traits under natural polymicrobial disease challenge
Source: Sci Rep. 2021 Oct 19;11:20628. doi: 10.1038/s41598-021-99778-9 (PMC8526711; doi:10.1038/s41598-021-99778-9)
Supplement: Supplementary file 4 — Supplementary Table S3. [file 41598_2021_99778_MOESM4_ESM.docx]

**Supplementary Table S3**. Estimates of genetic (above the diagonal) and phenotypic (below the diagonal) correlations (SE in parentheses) among betaine, dimethylglycine, citric acid and L-Glycine. Significance of genetic and phenotypic correlations are indicated as: **, *, corresponding to P<0.01 and P<0.05 respectively. “-” indicates not estimable.

| Metabolite | Betaine | Dimethylglycine | Citric acid | L-glycine |
| --- | --- | --- | --- | --- |
| Betaine | 1 | 0.28 (0.14) | 0.09 (0.25) | 0.19 (0.19) |
| Dimethylglycine | 0.40 (0.02)** | 1 | 0.16 (0.21) | 0.164 (0.22) |
| Citric acid | 0.29 (0.03) | 0.09 (0.04) | 1 | -0.17 (0.23) |
| L-glycine | 0.33 (0.04)* | 0.09 (0.04) | 0.18 (0.38) | 1 |
