# Supplementary material for: Heritability and genetic correlations of plasma metabolites of pigs with production, resilience and carcass traits under natural polymicrobial disease challenge
Source: Sci Rep. 2021 Oct 19;11:20628. doi: 10.1038/s41598-021-99778-9 (PMC8526711; doi:10.1038/s41598-021-99778-9)
Supplement: Supplementary file 5 — Supplementary Table S4. [file 41598_2021_99778_MOESM5_ESM.docx]

**Supplementary Table S4**. Descriptive statistics of plasma metabolites and two amino acid indexes: branched amino acids (BAA) and ketogenic aminoacids (ketoAA) in 958 animals

| **Metabolite (μM)** | **Minimum** | **Maximum** | **Mean** | **SD** | **CV** | **Range** |
| --- | --- | --- | --- | --- | --- | --- |
| 1-Methylhistidine | 3.70 | 20.92 | 10.68 | 3.34 | 0.31 | 17.22 |
| 2-Hydroxybutyrate | 0.76 | 2.99 | 1.28 | 0.25 | 0.19 | 2.23 |
| Betaine | 8.66 | 204.19 | 76.65 | 32.32 | 0.42 | 195.53 |
| Acetoacetate | 0.75 | 10.75 | 4.18 | 1.91 | 0.46 | 10.00 |
| Creatine log | 0.85 | 2.75 | 1.65 | 0.34 | 0.20 | 1.89 |
| Dimethylglycine | 1.19 | 19.62 | 5.86 | 3.46 | 0.59 | 18.43 |
| Citric acid | 32.40 | 380.52 | 201.13 | 53.91 | 0.27 | 348.12 |
| Ethanol log | -0.10 | 2.54 | 1.13 | 0.38 | 0.34 | 2.68 |
| D-glucose | 10.00 | 6132 | 2629.81 | 1266.36 | 0.48 | 6122 |
| L-glycine | 333.00 | 2904 | 1351.40 | 405.84 | 0.30 | 2571 |
| Glycerol | 51.00 | 554 | 238.28 | 81.14 | 0.34 | 503 |
| Formate | 0.82 | 2.89 | 2.06 | 0.46 | 0.22 | 2.07 |
| L-glutamic acid | 77 | 933 | 386.71 | 123.69 | 0.32 | 856 |
| Hypoxanthine | 44 | 293 | 161.74 | 39.19 | 0.24 | 249 |
| L-tyrosine | 20.05 | 140.36 | 66.26 | 17.70 | 0.27 | 120.31 |
| L-phenylalanine | 21.99 | 132.13 | 67.86 | 13.26 | 0.19 | 110.14 |
| L-alanine | 187.00 | 1835 | 881.29 | 218.76 | 0.25 | 1648.00 |
| L-proline | 68.00 | 554 | 278.22 | 73.61 | 0.26 | 486.00 |
| L-threonine | 54.00 | 1232 | 406.22 | 209.42 | 0.52 | 1178.00 |
| L-asparagine | 6.80 | 119.98 | 48.77 | 18.01 | 0.37 | 113.18 |
| Isoleucine | 9.90 | 197.60 | 48.48 | 30.26 | 0.62 | 187.70 |
| L-histidine | 1.58 | 69.30 | 23.66 | 11.87 | 0.50 | 67.72 |
| L-lysine | 45.00 | 601.00 | 258.03 | 84.90 | 0.33 | 556.00 |
| L-serine | 32.00 | 383.00 | 183.54 | 51.22 | 0.28 | 351.00 |
| L-lactic acid | 3592 | 22497 | 10054.65 | 2675.98 | 0.27 | 18905.00 |
| L-aspartate | 4.27 | 62.16 | 25.68 | 7.98 | 0.31 | 57.89 |
| L-acetylcarnitine | 6.94 | 63.98 | 24.03 | 5.85 | 0.24 | 57.04 |
| Oxoglutarate | 17.94 | 182.39 | 74.26 | 21.87 | 0.29 | 164.45 |
| L-ornithine | 18.00 | 295.00 | 93.77 | 45.84 | 0.49 | 277.00 |
| Pyruvic acid | 78.00 | 675.00 | 365.77 | 101.60 | 0.28 | 597.00 |
| Succinate | 2.56 | 77.76 | 28.78 | 10.16 | 0.35 | 75.20 |
| 3-hydroxybutyric acid | -0.60 | 1.77 | 0.48 | 0.23 | 0.48 | 2.37 |
| 2-hydroxyisovalerate | 0.30 | 6.28 | 1.17 | 0.62 | 0.52 | 5.98 |
| L-alpha-aminobutyric acid | 0.58 | 2.02 | 1.33 | 0.27 | 0.20 | 1.44 |
| 3-methyl-2-oxovaleric acid | 0.55 | 14.89 | 4.71 | 2.67 | 0.57 | 14.34 |
| L-arginine | 7.40 | 99.32 | 25.10 | 13.64 | 0.54 | 91.92 |
| Creatinine | 27.25 | 146.43 | 82.38 | 14.43 | 0.17 | 119.18 |
| L-glutamine | 58.00 | 900.00 | 371.52 | 115.96 | 0.31 | 842.00 |
| L-leucine | 28.00 | 230.00 | 108.49 | 27.83 | 0.25 | 202.00 |
| L-methionine | 4.49 | 116.25 | 40.39 | 19.50 | 0.48 | 111.76 |
| L-valine | 55.00 | 389.00 | 200.39 | 48.68 | 0.24 | 334.00 |
| Acetone | 0.42 | 15.89 | 2.44 | 1.41 | 0.58 | 15.47 |
| Isobutyric acid | 1.07 | 19.50 | 5.70 | 2.21 | 0.39 | 18.43 |
| Methanol | 0.66 | 2.58 | 1.74 | 0.29 | 0.17 | 1.92 |
| KetoAA | 88.00 | 734.00 | 366.51 | 95.87 | 0.26 | 646.00 |
| BAA | 104.43 | 719.69 | 357.36 | 87.88 | 0.24 | 615.26 |
